# Supplementary material for: Genetic diversity of Toscana virus glycoproteins affects the kinetics of virus entry and the infectivity of newly produced virions
Source: Npj Viruses. 2025 Apr 15;3:28. doi: 10.1038/s44298-025-00113-0 (PMC12000347; doi:10.1038/s44298-025-00113-0)
Supplement: Supplementary file 1 — Thiesson_et_al_2025_R1_Supplementary_Information_v2 [file 44298_2025_113_MOESM1_ESM.pdf]

Genetic diversity of Toscana virus glycoproteins affects the kinetics of virus entry and the infectivity of newly produced virions

Adrien Thiesson[1], Marie-Pierre Confort[1], Sophie Desloire[1], Alain Kohl[2,3], Frédérick Arnaud[1*], Maxime Ratinier[1*]

[1]IVPC UMR754, INRAE, Universite Claude Bernard Lyon 1, EPHE, Université PSL, F-69007 Lyon, France.

[2]MRC-University of Glasgow Centre for Virus Research, Glasgow, UK.

[3]Centre for Neglected Tropical Diseases, Departments of Tropical Disease Biology and Vector Biology, Liverpool School of Tropical Medicine, Liverpool, UK.

*Corresponding authors: frederick.arnaud@univ-lyon1.fr and maxime.ratinier@univ-lyon1.fr

Supplementary Information

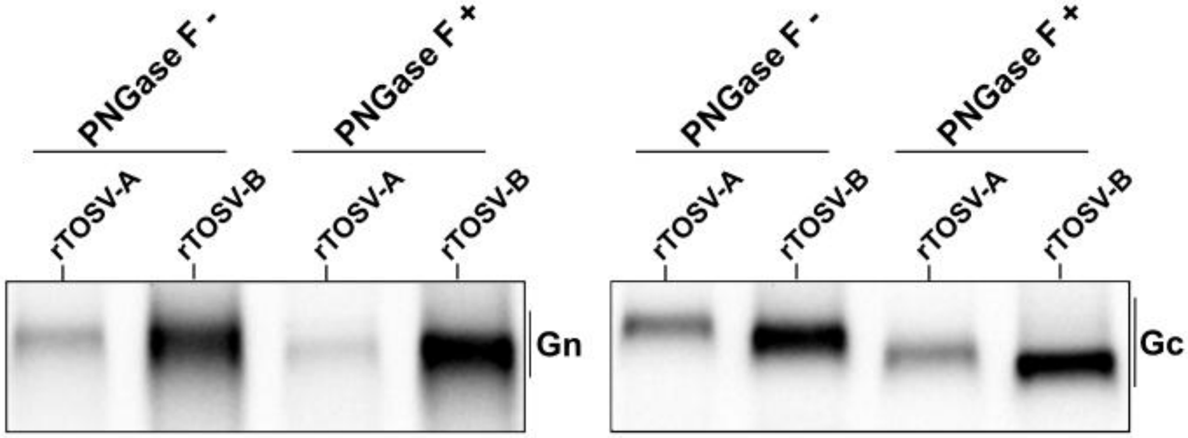

Fig S1. N-glycosylation pattern of rTOSV-A and rTOSV-B Gn and Gc glycoproteins. rTOSV-A and rTOSV-B virions produced from A549 Npro infected cells were purified by ultracentrifugation and treated or not with PNGase-F. The virions were then analysed by western blot with antisera raised against TOSV Gn or Gc proteins as indicated. Note that there is still a difference in the size of rTOSV-A and rTOSV-B Gn glycoproteins even after the PNGase-F treatment.

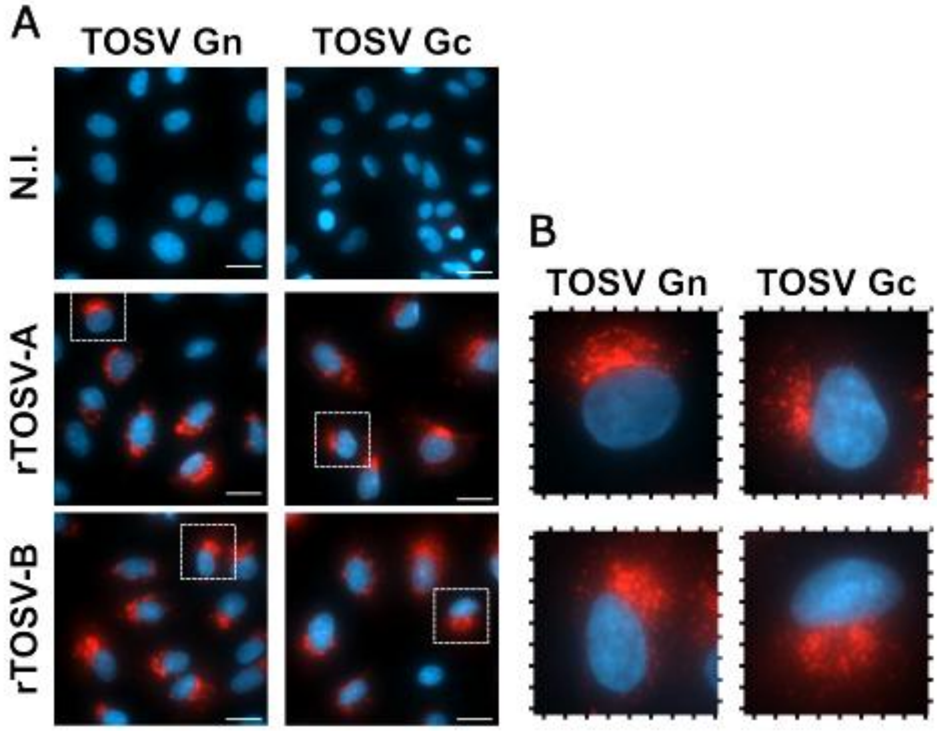

Fig S2. Cellular localization of rTOSV-A and -B Gn and Gc glycoproteins. (A) A549 Npro cells were either non-infected (N.I.) or infected with rTOSV-A or rTOSV-B viruses (MOI=0.3). At 14 hours post-infection, the cells were fixed with PFA and immuno-stained for TOSV glycoproteins with an anti-Gn or anti-Gc antibody followed by a secondary antibody conjugated to Alexa568 (in red). Cell nuclei were labelled with DAPI staining (in blue). Representative pictures of TOSV-infected cells from two independent experiments are shown. The scale bars at the low right corner represent 20 µm. (B) Magnification of areas are indicated with white squares.

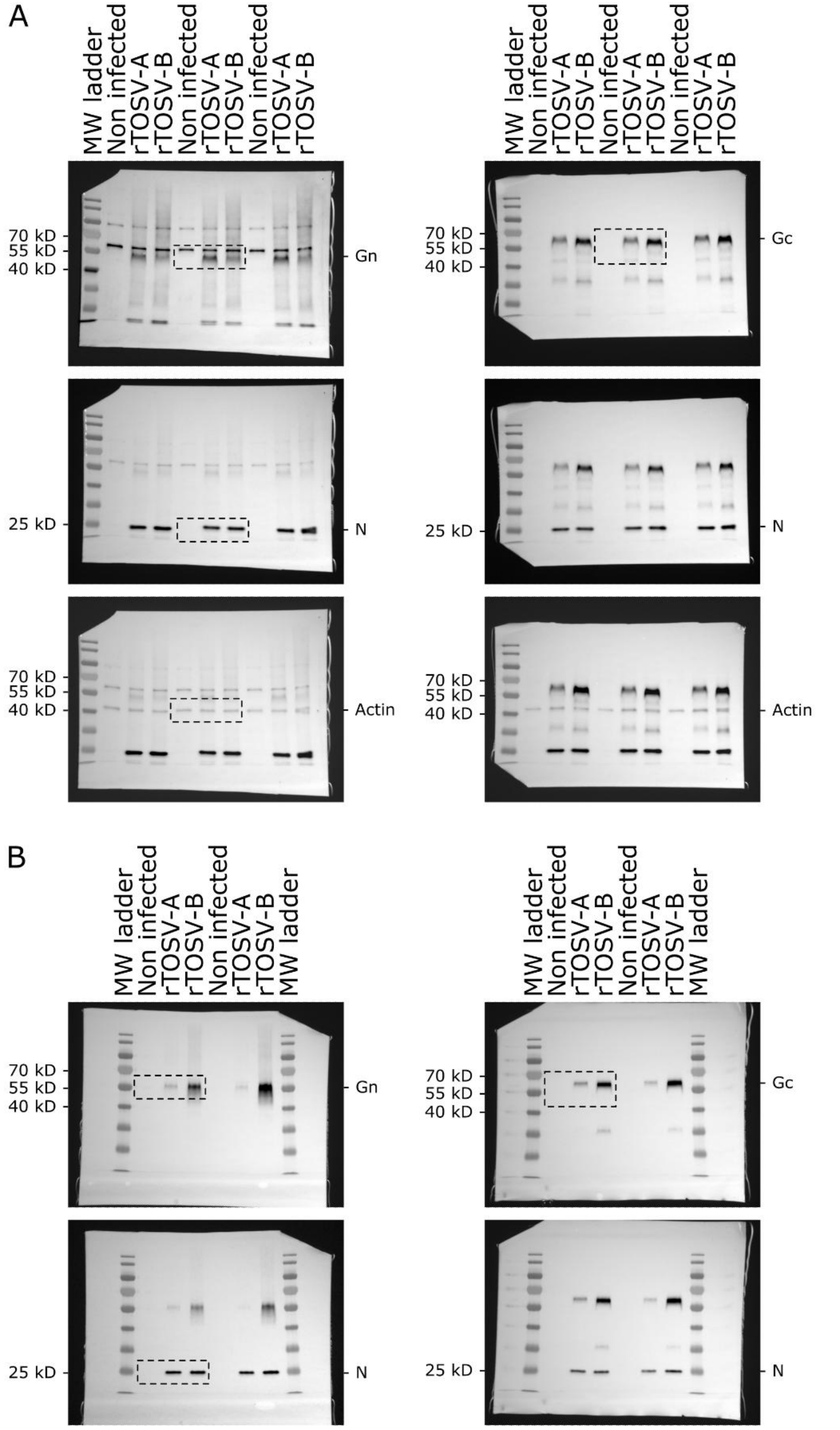

Fig S3. Uncropped western blot images. Uncropped images of western blots shown in Fig 6A. (A) Intracellular. (B) Extracellular.
